# A Rank Offence

**Published:** 1876-09

**Authors:** 


There is among us a large class of men and boys who indulge in a terribly unclean and offensive practice in the very presence of other persons, and who do not seem to be aware of the offence they give to cleanly citizens. A great army of persons labor day after day at the disgusting work of rolling into cylindrical form a certain noxious weed, the flavor and smell of which quickly sicken a person not accustomed to it. These rolled weeds are used by others for burning, the smoke from them being drawn into the mouth, and not infrequently forced out through the nose. Of course the result is to make the person who is compelled to swallow clouds of this vile smoke terribly offensive to all who come near him. The pungent creosote-like vapor penetrates the delicate mucus membrane of the mouth, nose, and fauces, and the breath exhaled over surfaces thus saturated is very unsavory. To be in close proximity to such a person is to be miserable indeed.

There is another numerous class for whom this same offensive and poisonous weed is prepared in different forms, and who are not content to burn it, but enjoy eating it. Generally they do not swallow much of it because the stomach is not willing to retain so destructive a poison. The common method is to roll it over in the mouth, saturate it with saliva, and as this fluid becomes darkened with the pungent extract, it is ejected from the mouth in the presence of others, on the floors of public conveyances, and in other public places. In fact, so common are these practices, that in some parts of the country vessels are placed to receive the saliva thus impregnated with poison, in order that carpets and floors may be spared from contamination!

These habits are not simply very unclean; they are very injurious as well. They probably never obtain a secure hold upon a human being without doing him a serious injury, and essentially shortening his life. There is a little organ in the chest which works away by night and by day, at all seasons, and under all circumstances. This muscular structure must keep up its regular contractions and expansions, or life will be suspended. Now, this weed we have mentioned, has a remarkable power to weaken the force of the heart's action. After a while an intermission occurs in the heart-beats, so that instead of seventy-five beats in a minute there will be but sixty or so, and this result will be reached, not by lengthening the spaces between the beats, but by failing to contract regularly. Thus there will be three, or four, or five, or six continuous beats, and then a pause. This is a kind of partial and incipient paralysis of the most vital organ, and for this variety of poisoning there is no cure. A person thus affected may live on for years, but the chances are that he will die early. Let this heart-paralysis proceed but a single step further so that two beats fail, that is, let the heart contract for the purpose of expelling the blood only once where it should do so three times, and death will soon follow.

There are two reasons, then, why a human being residing among other human beings, should not smoke or chew this vile weed. The first reason is, because it makes him fearfully offensive to all cleanly beings. The second reason is because the use of this potent poison impairs the health and endangers life.

